# Laparoscopic sigmoidectomy postopen aortic replacement for abdominal aortic aneurysm: a case report

**DOI:** 10.1097/MS9.0000000000000519

**Published:** 2023-04-06

**Authors:** Goshi Fujimoto, Takashi Deguchi

**Affiliations:** Department of Gastroenterological Surgery, Koga Community Hospital, Yaizu, Shizuoka, Japan

**Keywords:** abdominal aortic aneurysm, aortic replacement, case report, laparoscopic sigmoidectomy

## Abstract

Colectomy for colorectal cancer after an open aortic replacement (OAR) for abdominal aortic aneurysms has high perioperative complication and mortality rates.

**Case presentation:**

The authors report the case of an 87-year-old man who underwent laparoscopic sigmoidectomy. The patient presented with edema of the lower legs and face, and blood test results indicated anemia. The patient had a history of OAR for an abdominal aortic aneurysm 9 years prior, a left common iliac artery aneurysm, and a jump bypass graft. A colonoscopy revealed a type 2 lesion in the sigmoid colon; he was diagnosed with moderately differentiated adenocarcinoma. Preoperative computed tomography did not show any obvious lymph node or distant metastases. Laparoscopic sigmoidectomy with D3 lymphadenectomy was planned. During surgery, the use of the lateral approach allowed sigmoid mesocolon mobilization while confirming the presence of the artificial arteries. As the approach to the root of the inferior mesenteric artery was difficult, D1 lymphadenectomy was performed. No evidence of anastomotic leakage or artificial artery infection was observed postoperatively.

**Clinical discussion:**

Intra-abdominal adhesions due to the prior OAR makes sigmoid mesocolon mobilization difficult. In cases where laminar structure cannot be recognized, other landmarks are needed.

**Conclusions:**

After OAR, artificial arteries can be used as landmarks during colectomy. Although laparoscopic surgery is technically challenging, the magnified view provides an advantage in identifying these landmarks. Patients’ surgical records for the previous OAR should be checked, and the positions of the vessels and ureters should be elucidated preoperatively using computed tomography.

## Introduction

Highlights

- Colectomy after open aortic replacement for abdominal aortic aneurysm has high perioperative complication and mortality rates.
- Postoperative intra-abdominal adhesions and ureter position changes are problematic.
- The vessel and ureter positions should be elucidated preoperatively using computed tomography.
- Artificial arteries can be used as landmarks for mobilizing the sigmoid mesocolon.

In some cases, colectomy for colorectal cancer (CRC) may be required after open aortic replacement (OAR) or endovascular aortic aneurysm repair (EVAR) for abdominal aortic aneurysm (AAA). Patients undergoing OAR have a higher perioperative complication and mortality rates than those undergoing EVAR^1^–^3^. Intestinal adhesions from prior OAR can cause difficulties during colectomy procedures and compromise perioperative outcomes^1^. Nevertheless, specific surgical precautions and procedural points for colectomy following OAR have not been established. Herein, we report a case wherein laparoscopic sigmoidectomy was performed for sigmoid colon cancer after OAR without postoperative anastomotic leakage or artificial artery infection to help establish safer procedures. This case has been reported in line with the SCARE 2020 criteria^4^.

## Presentation of case

**Figure 1:**
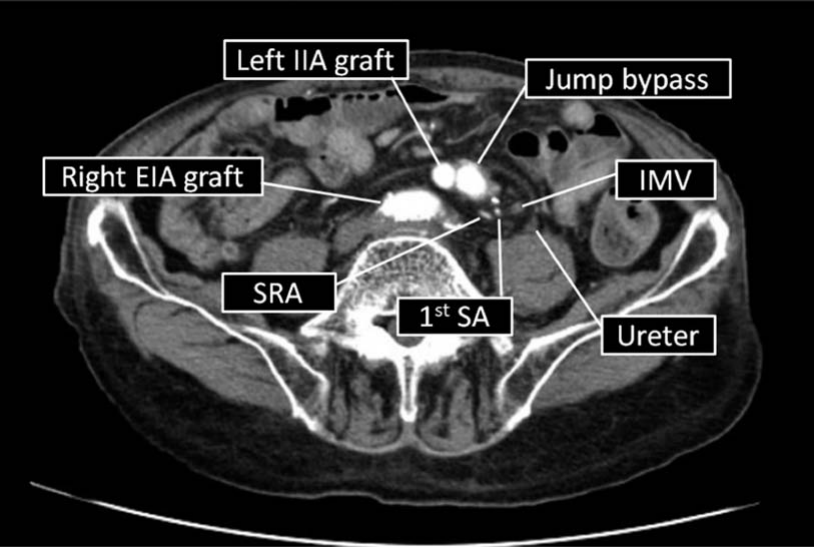
Findings on an arterial phase contrast-enhanced computed tomography image. The image reveals the jump bypass and external iliac artery (EIA) graft on the ventral side of the superior rectal artery (SRA). IIA, internal iliac artery; IMV, inferior mesenteric vein; SA, sigmoid artery.

An 87-year-old man was admitted to our hospital for edema of the lower legs and face. He had a medical history of OAR for AAA and a left common iliac artery aneurysm (CIAA) 9 years previously. The AAA was located below the renal artery. The abdomen was opened through a midline abdominal incision. The material of the artificial vessel used was unknown. Four-branched artificial artery (diameters of the main and branch ducts were 18, 9, and 8 mm, respectively) was used. The material of the graft used for jump bypass was also unknown, and the use of autovein graft was not mentioned. During the previous OAR, the sigmoid mesocolon was incised to repair the left CIAA, the inferior mesenteric artery (IMA) was ligated, and a jump bypass from the graft to the left common femoral artery was added due to a thrombus in the left external iliac artery graft. Blood examination results showed anemia (hemoglobin, 10.1 g/dl), but normal serum levels of carcinoembryonic antigen (2.1 ng/ml; reference range: 0–5 mg/ml) and carbohydrate antigen (6.2 U/ml; reference range: 0–37 U/ml). A colonoscopy revealed a type 2 lesion in the sigmoid colon, and the condition was diagnosed as moderately differentiated adenocarcinoma. Contrast-enhanced computed tomography (CECT) demonstrated a thickened sigmoid colon wall, which indicated a type 2 lesion without obvious lymph nodes or distant metastases. Based on the eighth edition of the UICC TNM classification^5^, the patient was diagnosed preoperatively with locally advanced sigmoid cancer (cT3N0M0, stage IIA). Arterial phase CECT indicated the jump bypass and external iliac artery graft were on the ventral side of the IMA and superior rectal artery (SRA) and on the dorsal side of the ureter (Fig. 1). The patient consented to laparoscopic surgery understanding that surgery would be challenging due to adhesions and high risk of postoperative complications.

**Figure 2:**
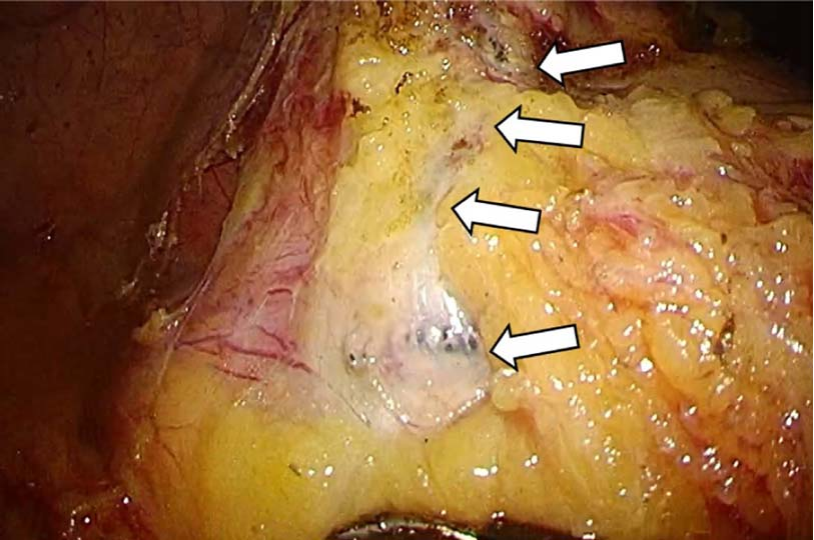
Intraoperative findings. The lateral approach allowed mobilization of the sigmoid mesocolon while exposing the artificial artery (arrows).

Laparoscopic sigmoidectomy with D3 lymphadenectomy was planned. The patient was placed in the lithotomy position under general anesthesia. A 12-mm camera port was inserted 1 cm below the umbilicus by the open method. Laparoscopic observation performed with the VISERA ELITE system and ENDOEYE FLEX (OLYMPUS LTF-S190-10) revealed no adhesion on the abdominal wall and no metastasis to the liver and peritoneum. After helping the patient in the head-low position, a 12-mm trocker was inserted 2 cm medial to the right superior anterior iliac spine. Next, 5-mm trockers were inserted 2 cm medial to the left superior anterior iliac spine, and 10 cm cranial to the 12 and 5-mm trocker inserted earlier. The medial approach allowed exposure of the rectal fascia propria caudal to the promontorium. However, the laminar structure could not be recognized in the vicinity of the artificial artery. The lateral approach permitted sigmoid mesocolon mobilization while confirming the ureter and artificial artery positions, which were observed through the retroperitoneum (Fig. 2). The ureter was lateral to its typical position. The artificial artery was a landmark for the sigmoid mesocolon border on the lateral side. Thereafter, the presence of the ventral side of the artificial artery was confirmed and partially exposed from the medial side of the sigmoid mesocolon. These lateral and medial approaches visualized the mesocolon area for D1 lymphadenectomy. Instead of the planned D3 lymphadenectomy, we performed D1 lymphadenectomy because the approach to the root of the IMA and SRA was difficult due to their location on the dorsal side of the artificial artery. After confirming the absence of any ischemic findings on the cutting margin of the sigmoid colon, end-to-end anastomosis with a double stapling technique was performed. Excessive tension at the anastomosis site was not observed. The operation time was 3 h 29 min, and the intraoperative blood loss was 175 ml.

**Figure 3:**
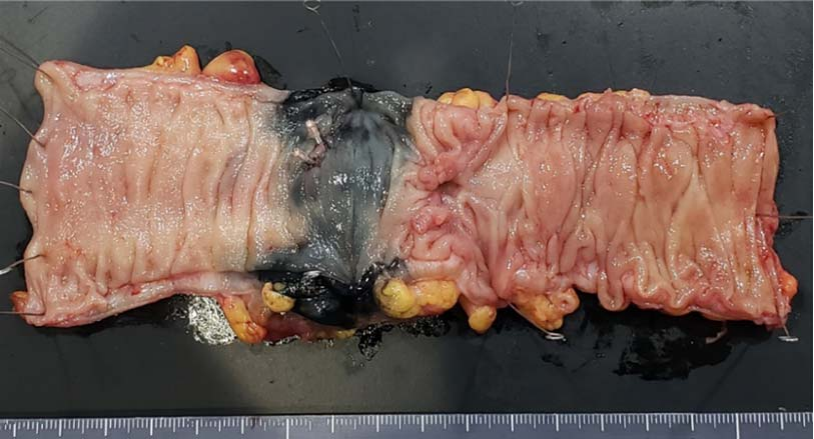
Macroscopic findings of the resected specimen. The specimen shows a type 2 lesion measuring 15×8 mm.

Macroscopic evaluation of the resected specimen revealed a type 2 lesion measuring 15×8 mm (Fig. 3). Histopathological examination indicated moderately differentiated adenocarcinoma of the sigmoid colon with mesenteric lymph node metastasis (pT3N1aM0, stage IIIB). The patient had no postoperative complications except for a coronavirus disease 2019 infection, which required isolation. The patient refused postoperative adjuvant chemotherapy owing to his general condition. No anastomotic leakage, artificial artery infection, or recurrence was observed at 6 months postoperatively.

## Discussion

Concomitant AAA and CRC are reported in 0.5–4% of AAA patients^2^, of whom 8.4–24.1% undergo colectomy after OAR^1^,^2^,^6^. As EVAR is not indicated for all AAA cases, colectomy for CRC after OAR is occasionally necessary, although the perioperative complication rate is high^7^. In such cases, the precautions regarding colectomy surgical techniques should be reviewed. Surgical techniques for sigmoidectomy include sigmoid mesocolon mobilization and lymphadenectomy. Colectomy is challenging, and outcomes after OAR are poor because of the long recovery time from OAR, with heavy bleeding and intra-abdominal adhesions post-OAR^1^. In our case, colectomy was performed 9 years following OAR. Despite sufficient recovery from OAR, the effects of intestinal adhesions were substantial in our patient. In the prior OAR, the retroperitoneum had been closed, which may have limited the development of intestinal adhesions. However, adhesion between the artificial artery and sigmoid mesocolon can make lymphadenectomy and sigmoid mesocolon mobilization difficult. Thus, the lateral approach is safer. The medial approach elucidates the SRA location by exposing the rectal fascia propria on the caudal side of the artificial artery. The boundary between the peritoneum and sigmoid mesocolon can be identified using mesocolon fatty tissue as a landmark. Care must be taken to avoid incising the peritoneum covering the artificial artery and causing ureteral damage on the ventral side of the artificial artery. The ureter’s position should be confirmed by preoperative CT because its original position may differ due to intraoperative manipulation during the former OAR. Medial to the ureter, the ventral side of the artificial artery is a landmark for mobilization of the mesocolon. Lymphadenectomy is difficult in sigmoidectomy because the artificial artery is close to the IMA, which is the landmark for D3 lymphadenectomy. As the IMA may be ligated or reconstructed during prior OAR, the IMA should be checked by medical record and CT preoperatively. If the sigmoid mesocolon is incised with an additional OAR for left CIAA or aortofemoral bypass, lymphadenectomy becomes more difficult. D3 lymphadenectomy is challenging in cases of aortofemoral bypass because the IMA root is located dorsal to the artificial artery. D2 lymphadenectomy is also difficult when the artificial artery penetrates the sigmoid mesocolon, and the SRA runs dorsal to the artificial artery. In such cases, D1 dissection confirming the presence of the ventral side of the artificial artery through the retroperitoneum is safe and allows identification of the sigmoid mesenteric penetration site and dissection of the sigmoid colon artery on its peripheral side. Identification of metal clips or staples placed during mesocolon dissection on postoperative CT images acquired during follow-up may help clarify anatomical positioning.

Post-OAR colectomies have a greater risk of graft infection than post-EVAR colectomies^7^. Prosthetic graft infections may lead to death^2^,^7^,^8^. A case of post-OAR left hemicolectomy for sigmoid colon cancer has been reported to cause sepsis by graft infection postoperatively, thereby leading to death^8^. To reduce the infection risk, the omentum is used to cover the artificial artery. In our case, the artificial artery was not covered, but it should have been covered by the omentum or the sigmoid mesocolon since it was partially exposed. As anastomotic leakage is also a risk factor for infection, a defunctioning stoma may be constructed^9^. Hartmann’s procedure should be considered if there is a sufficient distance between the sigmoid colon and rectum, considering the anastomotic leakage risk due to excessive tension at the anastomosis site. Given that the sigmoid mesocolon adheres to the artificial artery when the artificial artery penetrates the sigmoid mesocolon, mobilization of the splenic flexure may not be effective for reducing tension at the anastomotic site. Although the effect of postoperative prophylactic antibiotic administration is unknown, antibiotics were only used intraoperatively and did not cause artificial vascular infection in our case.

Ureteral injury occurs in 0.3–1.5% of colon resection surgeries^10^,^11^. It is reasonable to prioritize the lateral approach since medial-to-lateral dissection (medial approach) has been identified as a risk factor for ureteral injury^12^. Although insufficient evidence exists to determine whether prophylactic ureteral stenting (PUS) in CRC significantly reduces ureteral injury^13^, PUS may help prevent and identify ureteral injury and ureteral repair^12^. The usefulness of a new ureteral catheter, the Near-Infrared Ray Catheter (NIRC) fluorescent ureteral catheter (NIRFUC), has also been reported, and PUS should have been considered in this case^10^.

In the present case, laparoscopic sigmoidectomy was technically challenging owing to a previous OAR, in that CIAA repair and a jump bypass had been added. In cases where the graft used in OAR is anastomosed to the bilateral common iliac artery, the surgical precautions and procedural points may be different.

## Conclusion

Sigmoidectomy after OAR for AAA is difficult due to adhesions. Patients’ surgical records for the previous OAR should be checked, and the positions of the vessels and ureters should be elucidated preoperatively using CT. This case report emphasizes the necessity of prioritizing the external approach during the surgical procedure and using artificial arteries as landmarks for mobilizing the sigmoid mesocolon. Furthermore, although laparoscopic surgery is technically challenging, the magnified view provides an advantage in identifying these landmarks. Further studies are warranted to improve the perioperative outcomes of colectomy following OAR.

## Ethical approval

All procedures followed were in accordance with the ethical standards of the responsible committee on human experimentation (institutional and national) and with the Helsinki Declaration of 1975, as revised in 2008(5). This case report was approved by the Research Ethics Committee of Koga Community Hospital, Japan (No. 2022-5).

## Consent

Written informed consent was obtained from the patient for the publication of this case report and accompanying images.

## Sources of funding

This research did not receive any specific grants from funding agencies in the public, commercial, or not-for-profit sectors.

## Author contribution

G.F. performed the operation, wrote the manuscript, and approved the final manuscript. T.D. assisted in the operation, revised the manuscript, and approved the final manuscript.

## Conflicts of interest disclosure

The authors declare that they have no conflicts of interest.

## Research registration unique identifying number (UIN)

This study was registered as a case report in the UMIN Clinical Trials Registry (https://www.umin.ac.jp/ctr/) with the unique identification number UMIN000049293.

## Guarantor

Goshi Fujimoto.

## Data availability statement

Data supporting the findings of this study are available in the supplementary material of this article.

## Provenance and peer review

Not commissioned, externally peer reviewed.
